# Yiqi Huazhuo decoction increases insulin secretion in type 2 diabetic rats by regulating the pancreatic GPR40-IP3R-1 signaling pathway

**DOI:** 10.3389/fphar.2023.1136778

**Published:** 2023-03-14

**Authors:** Dongjiao Wu, Siying Weng, Shuyi Xu, Yan Li, Jianyang Zhou

**Affiliations:** ^1^ Department of Rheumatology, Ningbo Municipal Hospital of TCM, Affliated Hospital of Zhejiang Chinese Medical University, Ningbo, China; ^2^ Department of Endocrinology, Ningbo Municipal Hospital of TCM, Affliated Hospital of Zhejiang Chinese Medical University, Ningbo, China; ^3^ College of the Third Clinical Medical, Ningbo Municipal Hospital of TCM, Affliated Hospital of Zhejiang Chinese Medical University, Ningbo, China

**Keywords:** Yiqi Huazhuo decoction, type 2 diabetes, pancreatic islet β-cell, impaired insulin secretion, free fatty acid receptor 1

## Abstract

**Objective:** Yiqi Huazhuo Decoction (YD) reduces blood glucose, glycated hemoglobin, body weight, and insulin resistance in patients with type 2 diabetes mellitus (T2DM), but its exact mechanisms are unknown. This study investigated the therapeutic effects and mechanisms of YD on impaired insulin secretion in T2DM rats.

**Methods:** T2DM rats were randomized to the model, YD-lo (15 mg/kg/d YD, 10 weeks), YD-hi (30 mg/kg/d YD, 10 weeks), positive drug (TAK-875), and healthy control groups. The rats underwent an oral glucose tolerance test (OGTT), glucose-stimulated insulin secretion (GSIS) test, and serum lipid measurements. High-fat and high-glucose-injured RIN-m5f cells were treated with YD (30 or 150 mg/mL) for 48 h. GPR40 and IP3R-1 expression levels were determined by immunofluorescence, qRT-PCR, and western blot.

**Results:** Compared with the model group, the OGTT area under the curve (AUC) in the YD-hi group was decreased by 26.7%, the insulin release test (IRT) AUC in the YD-hi group was increased by 45.9%, and the GSIS AUC was increased by 33.9% (*p* < 0.05). Compared with the model cells, the insulin secretion after glucose stimulation in the YD-hi group was increased by 24.5%, similar to the TAK-875 group (23.1%) (*p* > 0.05). GPR40 and IP3R-1 mRNA in the model cells were decreased by 49.5% and 51.2% compared with the control cells (*p* < 0.05). In the YD-hi group, GPR40 and IP3R-1 mRNA levels were increased by 58.1% and 39.3% (*p* < 0.05), similar to the TAK-875 group. The changes in protein expression were similar to mRNA.

**Conclusion:** YD promotes insulin secretion from pancreatic islet β-cell in T2DM rats by regulating the GPR40-IP3R-1 pathway, thereby reducing blood glucose.

## Introduction

Diabetes mellitus (DM) is a chronic disease with a high incidence. According to the International Diabetes Federation (IDF) Diabetes Atlas (10th Edition), as of 2021, 537 million adults aged 20-79 (10.5%) have diabetes, and China has the largest number of diabetics in the world. By the end of 2017, the number of adults with DM had reached 116 million in China, accounting for 11.2% of the national population (Li et al., 2020). The potential complications of DM include cardiovascular disease, neuropathy, nephropathy, retinopathy, and increased mortality (American diabetes Association Association, 2014). As the global population is aging, an increasing proportion of people over the age of 60 will have diabetes, placing a large burden on the economy (Sharma et al., 2017; Sun et al., 2022; Wang et al., 2022).

Type 2 diabetes mellitus (T2DM) is characterized by variable degrees of insulin resistance and deficiency, resulting in hyperglycemia (American diabetes Association, 2022, 2014; Chatterjee et al., 2017). T2DM accounts for more than 90% of DM cases (Tang et al., 2019). The global prevalence of T2DM is estimated at 9.0% in men and 7.9% in women (Collaboration, 2016). T2DM is the product of the interplay between decreased insulin secretion caused by pancreatic islet β-cell dysfunction and peripheral resistance to insulin action, leading to decreased glucose uptake. Currently, drugs used to treat type 2 diabetes include metformin, insulin secretagogues, α-glycosidase inhibitors, thiazolidinediones (TZD), dipeptidyl peptidase IV inhibitors (DPP-4i), sodium-glucose cotransporter 2 inhibitors (SGLT2i) glucagon-like peptide 1 receptor agonist (GLP-1RA), and insulin (Sharma et al., 2018a; American diabetes Association, 2022). Both sulfonylureas and non-sulfonylureas antidiabetic drugs are insulin secretagogues that directly act on pancreatic islet β-cell, while G-protein-coupled receptor 40 (GPR40) agonists have different targets on β-cell as new insulin secretagogues.

The pancreatic islet β-cell transmembrane protein GPR40 (also named the free fatty acid receptor 1 (FFAR1)) belongs to the G protein-coupled receptor family and is a natural receptor for medium- and long-chain fatty acids. GPR40 is mainly distributed in the cell membrane of islet β-cells in the human pancreas and is a transmembrane protein that is characteristically highly expressed in β-cells (Ichimura et al., 2009). GPR40 can trigger the extracellular secretion of insulin after releasing Ca^2+^ from the endoplasmic reticulum (ER) through the phospholipase C (PLC) pathway under the stimulation of free fatty acids (FFAs) (Shapiro et al., 2005; Feng et al., 2012; Usui et al., 2019). Nevertheless, chronically elevated FFAs can decrease GPR40 expression, insulin biosynthesis, and glucose-stimulated insulin secretion and cause β-cell dysfunction (Chueire and Muscelli, 2021). In addition, 20-hydroxy-eicosatetraenoic acid (20-HETE) can activate GPR40 and form a positive feedback loop to enhance glucose-stimulated insulin secretion (Tunaru et al., 2018). GPR40 can affect palmitate-stimulated insulin secretion by enhancing mitochondrial respiration (Kristinsson et al., 2015). GPR40 is involved in the FFA-mediated glucose-dependent insulin secretion (GDIS) effect of β cells but not in the chronic toxicity of FFAs. It is an important non-traditional target for promoting insulin secretion (Yashiro et al., 2012). The activation of GPR4 regulates glucose-stimulated insulin secretion in the high-fat obesity state (Schnell et al., 2007). Therefore, modulating the activity of GPR40 might be an attractive way to increase insulin secretion (Feng et al., 2012).

For centuries, herbal drugs have been widely used as complementary and alternative medicine (CAM) to treat chronic diseases such as diabetes. In recent decades, more and more diabetes patients (72.8%) have used herbs, dietary supplements, and other CAM therapies (Chang et al., 2007). Currently, many medicinal plants have been used to treat DM and its related conditions (Qi et al., 2010; Mao et al., 2021; Mao et al., 2022), of which herbs such as Yunpi Heluo decoction and dietary supplements can be used to treat DM.

Yiqi Huazhuo Decoction (YD) is a traditional Chinese medicine (TCM) preparation consisting of four herbs: Radix Astragali (*Astragalus mongholicus* Bunge (Fabaceae)), Atractylodes Rhizoma (*Atractylodes lancea* DC), Salviae Miltiorrhizae Radix et Rhizoma (*Salvia miltiorrhiza* Bunge), and Radix Puerariae Lobatae (*Pueraria montana var. lobata* (Willd.) Maesen & S.M.Almeida ex Sanjappa & Predeep). The plant names have been checked with https://wfoplantlist.org/plant-list. YD has been used for a long time in treating T2DM with mildly impaired pancreatic islet function (Gong et al., 2013). In a previous multicenter randomized controlled clinical trial on the use of YD for the treatment of T2DM, it was found that YD could reduce blood glucose, glycated hemoglobin (HbA1c), body weight, and insulin resistance in overweight patients with T2DM (Chen et al., 2013). It was also observed *in vitro* that YD could improve insulin secretion by decreasing the expression of miR-124-3p and decreasing the inhibition of the PLC-B1/IP3R-1 pathway in INS-1 cells (Weng et al., 2019). Nevertheless, how YD modulates insulin secretion by damaged pancreatic islet β-cells and whether GPR40 is involved remain unknown.

Therefore, this study investigated the regulatory mechanism of YD on insulin secretion of pancreatic islet β-cell in T2DM rat models. The results could provide more insights into the mechanisms of YD and provide a scientific rationale for its clinical use. In addition, it could provide additional data on the mechanisms of insulin secretion in T2DM.

## Materials and methods

### Preparation of YD

Radix Astragali (lot no. 20108) from Gansu Province of China, Atractylodes Rhizoma (lot no. 20115) from Liaoning Province of China, Radix Puerariae Lobatae (lot no. 20117) from Jiangxi Province of China, and Salviae Miltiorrhizae Radix et Rhizoma (lot no. 20201) from Zhejiang Province were purchased from Zhejiang University of Traditional Chinese Pharmaceuticals Co., Ltd. (http://www.zjzyyp.com/Default.aspx); all were inspected based on the Pharmacopoeia 2015 edition and the enterprise standard TS-ZL-03. YD comprises Radix Astragali 30 g, Atractylodes Rhizoma 30 g, Radix Puerariae Lobatae 30 g, and Salviae Miltiorrhizae Radix et Rhizoma 20 g. After the medicines were crushed, 550 g of water was added for decoction for 1 h. The filtrate was adjusted to pH 7.0 using sodium citrate and sodium carbonate. The obtained YD had a crude drug content of 3 g/mL, determined by a colorimetric method. The YD liquid was added to pure water, placed in a freeze-drying box, pre-frozen at −50°C for 4 h in a medicinal vacuum freeze dryer (TA, lyo_0.5), dried, sublimated at −20°C for 12 h, and dried at 32°C for 8 h. The YD freeze-dried powder was obtained, which was refrigerated at 4°C for later use. Before use, the powder was dissolved in the corresponding medium to the required concentration, centrifuged at 4000 *×g* for 30 min to remove the precipitate, and the supernatant was used.

### Chemical analysis of YD by UHPLC-Q-Orbitrap HRMS

**FIGURE 1:**
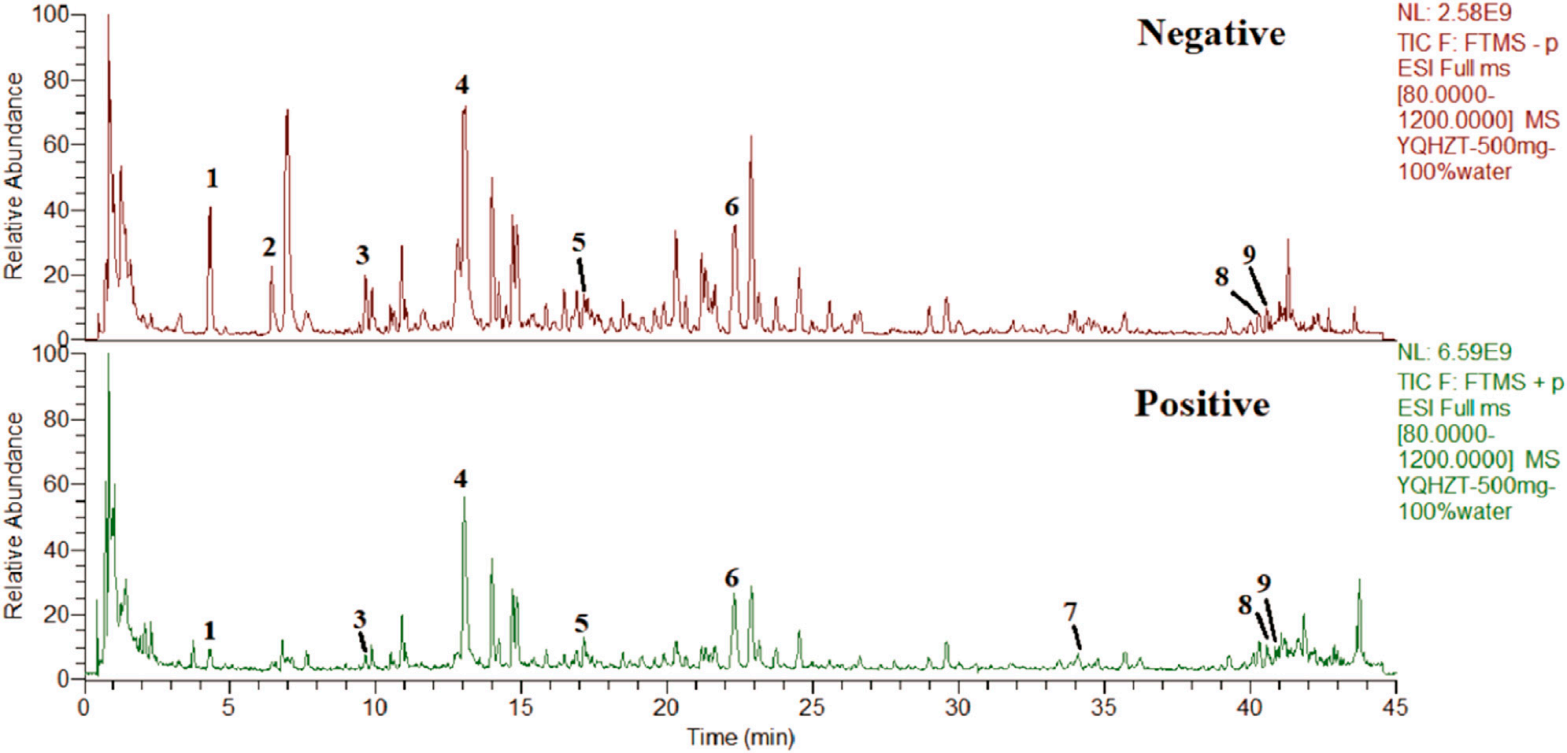
Total ion chromatograms of YD UHPLC-Q-Orbitrap HRMS in negative and positive ion modes. Salvianolic acid A (1), protocatechualdehyde (2), chlorogenic acid (3), puerarin (4), calycosin-7-O-β-D-glucopyranoside (5), salvianolic acid B (6), atractylodes Lactone I (7), astragaloside I (8), and astragaloside IV (9).

The chemical analysis of YD was carried out by ultra-high performance liquid chromatography-Q precision hybrid quadrupole orbitrap high-resolution mass spectrometry (UHPLC-Q-Orbitrap HRMS, Thermo Fisher Scientific Inc., Grand Island, NY, USA). UHPLC (Thermo Scientific Dionex Ultimate 3000) was controlled with the Chromeleon 7.2 software. The autosampler was set to 10°C and protected from light. The column was set to 40°C. A Waters ACQUITY UPLC BEH C18 Column (2.1 × 100 mm, 1.7 μm) was used. The mobile phase consisted of A (0.1% formic acid) and B (methanol) at a flow rate of 0.3 mL/min, gradient elution: 0–4 min (4% B), 4–10 min (4%–12% B), 10–30 min (12%–70% B), 30–35 min (70% B), 35–38 min (70%–95% B), 38–42 min (95% B), 42–45 min (95% B) −4% B). The injection volume was 2 μL. The mass spectrometer Q-Orbitrap system was connected to the UHPLC system *via* heated electrospray ionization and controlled by the Xcalibur 4.1 software for data acquisition and analysis. The electrospray ionization source was run and optimized under positive and negative ionization models. The mass spectrometry parameters were capillary temperature: 320°C; sheath gas (N2) flow rate: 35 arbitrary units; auxiliary gas (N2) flow rate: 13 arbitrary units; purge gas flow rate: 0 arbitrary units; spray voltage: 2.8 kV (negative) and 3.2 kV (positive); S lens RF level: 50 V; auxiliary gas heater temperature, 300°C; scan mode: full MS; scan range: 80–1,200 m/z. The maximum injection time (IT) was 200 ms. The scan resolution was 70,000 FWHM (m/z/s). The automatic gain control (AGC) target was 1.0e6. The typical chromatographic fingerprint of the YD extract is shown in Figure 1.

### Animal models

Thirty-two specific pathogen-free (SPF) 8-week-old male ZDF (fa/fa) rats, weighing 200–220 g, and eight SPF 8-week-old male ZL (fa/+) rats, weighing 200–220 g, were purchased from Beijing Weitong Lihua Laboratory Animal Technology Co., Ltd. (Beijing Viton Lever Laboratory Animal Company, Beijing, China). The animals were housed in the SPF experimental rearing room at the Animal Experiment Center of Zhejiang University of Traditional Chinese Medicine. All animal studies were approved by the Institutional Animal Care Committee of Zhejiang University of Traditional Chinese Medicine (#2017A610167). All experiments were performed with humane care according to the 3R principles (Hubrecht and Carter, 2019) and in accordance with the Association for the Assessment and Accreditation of Laboratory Animal Care (AAALAC) (https://www.aaalac.org/) and the Institutional Animal Care and Use Committee (IACUC) guidelines.

All rats were acclimatized for 1 week. The ZDF rats were fed a high-glucose and high-fat diet (4.0% crude fiber, 7.55% total fat, 23.75% protein, and digestible energy of 14.6 kJ/kg) for 4 weeks, while the control ZL rats were fed a normal diet for 4 weeks. A fasting blood glucose ≥11.1 mmol/L was used as the success criterion for establishing T2DM rats. After successful modeling, the ZDF rats were randomly divided into four groups, with eight rats in each group. Referring to a previous study (Gong et al., 2013), the doses of YD in the high-concentration (YD-hi) and low-concentration (YD-lo) groups were 30 and 15 g/kg, respectively. The positive control group was given 10 mg/kg TAK-875 (No.A8339, APE x BIO, Houston, USA) according to previous studies (Negoro et al., 2010; Leifke et al., 2012). The model group was given the same amount of distilled water once a day for 8 weeks. The study drugs (including the distilled water) were given by gavage. The ZL rats were fed with normal chow for 8 weeks as a control group.

Before the end of the experiment, the rats in each group were fasted for 12 h, euthanized with pentobarbital, and blood was collected by cardiac puncture. The abdominal cavity was quickly cut open, and the pancreas was taken out. Part of the pancreatic tissue was taken and fixed in a 10% formalin solution to prepare sections.

### Body weight, blood glucose, oral glucose tolerance test (OGTT), insulin release test (IRT), glucose-stimulated insulin secretion (GSIS) test, and serum lipid test

After successful modeling, each rat was weighed weekly, blood samples were collected from the tail vein once a week, and the fasting blood glucose level was measured using glucose test strips.

The OGTT test and IRT were conducted every 2 weeks. For the OGTT, the rats were fasted for 12 h, and the blood was collected from the tail vein. The glucose oxidase method (Toecho Super 2, Kagawa, Japan) was used to test the plasma fasting blood glucose levels. At the same time, 0.2 mL of blood was collected, the serum was collected after centrifugation, and the fasting insulin levels were determined by ELISA. Each group was given the corresponding drugs or distilled water for 30 min to measure the blood glucose and insulin levels, then the rats were given 20% glucose by gavage at 10 mL/kg body weight, and the glucose dose was 2 g/kg. Blood glucose and insulin were measured at 30, 60, 120, and 180 min after glucose administration. The area under the curve (AUC) of blood glucose and insulin was observed at each time point after glucose administration in each group. The formula was AUC = (0 min + 30 min × 0.25 + (30 min + 60 min) × 0.25 + (60 min + 90 min) × 0.5 + (90 min + 120 min) × 0.5.

For the GSIS, the rats were fasted for 12 h and anesthetized by an intraperitoneal injection of 3% pentobarbital (45 mg/kg) (Sharma K. et al., 2018; Sharma et al., 2018c). The rats were immobilized in the supine position, the right jugular vein was cannulated, and 50% glucose 0.5 g/kg was infused. At 0, 3, 5, and 10 min, 0.5 mL of blood was collected. After centrifugation at 5,000 r/min for 15 min, the supernatant was collected, and insulin was measured by ELISA. During the experiment, normal saline was injected to maintain the blood volume of the rats.

The rats were sacrificed after fasting for 12 h. Blood was collected from the heart, and serum total cholesterol (CHOL) and triglyceride (TG) levels were detected using a U2700 automatic biochemical analyzer (Hitachi, Tokyo, Japan) using the manufacturer’s reagents and instructions.

### Histopathological examination of the pancreas and pathological scores

The rat pancreatic tissues were fixed in 10% formalin for 5 days. The specimens were dehydrated with ethanol, treated with xylene for 30 min, and placed in paraffin for 5 h. The paraffin blocks were sectioned at 4 μm thickness. The paraffin sections were dewaxed with xylene, rehydrated with ethanol at 100%, 95%, 85%, and 70%, stained with hematoxylin, treated with 0.2% ammonia, and stained with 0.5% eosin. After ethanol dehydration and xylene transparency, the sections were sealed with neutral gum. The histopathological changes in the pancreas were observed under light microscopy. The nuclei were blue, the cytoplasm was pink, and the erythrocytes were orange-red. The Schmidt pathology scale was used to perform the pathological score of pancreas (Schmidt et al., 1992). The score was based on the degree of tissue edema, necrotic area, bleeding and inflammatory cell infiltration.

### Immunofluorescence for GPR40

The paraffin sections were dewaxed and rehydrated with xylene I for 20 min, xylene II for 20 min, 100% ethanol I for 5 min, 100% ethanol II for 5 min, 95% ethanol for 5 min, 80% ethanol for 5 min, and PBS three times, 3 min/time. The sections were placed in 0.01 M citrate buffer (pH 6.0) for antigen retrieval in a microwave, cooled to room temperature naturally, and washed with PBS three times, 3 min/time. The primary anti-rabbit polyclonal GPR40 antibody (1:50; C02655F; SAB Biotherapeutics, Sioux Falls, SD, USA) was incubated overnight at 4°C in a refrigerator, transferred to room temperature to equilibrate for 30 min, and washed with PBS three times, 5 min/time. Fluorescent secondary antibody donkey anti-rabbit antibody (1:300; Alexa Fluor® 488 donkey anti-Rabbit IgG secondary antibody; Life Technologies Co., Grand Island, NY, USA) was incubated at 37°C for 60 min, rinsed with PBS three times, 5 min/time. The nuclei were stained with DAPI (Abcam, Cambridge, United Kingdom) at room temperature for 10 min. After sealing with glycerol PBS sealer (IH0271; Shanghai Ruji Biotechnology Company, Shanghai, China), the sections were observed using a laser confocal microscope (Model 800, Carl Zeiss GmbH, Oberkochen, Germany).

### Cell culture

RIN-m5f cells were purchased from the National Collection of Typical Cell Cultures (Shanghai, China). The RINm5f cell line was inoculated in a 5.6 mmol/L glucose low-glucose DMEM medium (11885084; GIBCO, Invitrogen Inc., Carlsbad, CA, USA), 10% FBS (GIBCO, Invitrogen Inc., Carlsbad, CA, USA) with 1% penicillin/streptomycin and incubated in 5% CO_2_ at 37°C with saturated humidity. The modeling was performed as previously described (Ji et al., 2017). The model group was added with 30 mmol/L glucose, and sodium oleate/sodium palmitate (Xi’an Kunchuang Technology Co., Ltd., Xi’an, China, SYSJKJ001) was added to the final concentration of 500 μM for 48 h. The control group was cultured in a low-glucose medium. The RINm5f cells were divided into five groups: YD low-dose group (YD-lo), YD high-dose group (YD-hi), positive control group (GPR40 agonist TAK-875 50 nmol/L), model group (equal amount of culture medium), and untreated RINm5f cells as control.

### Cell counting kit-8 (CCK-8)

The effect of different concentrations of YD on cell proliferation was determined by the CCK8 assay. YD freeze-dried powder was added in a gradient of crude drug content of 0.01%, 0.1%, 1%, 5%, 10%, and 100%. The optimum concentration and time (24, 48, and 72 h) were screened for the subsequent experiments. The RIN-m5f cells in the logarithmic growth phase were adjusted to 2.5 × 10^7^/L and inoculated in a 96-well plate. After the cells adhered, the serum-free medium was replaced, and the culture was continued for 12 h. The cells were treated as above for 48 h. Then, 10 μL of CCK8 detection solution (UNOCI, China) was added to each well and incubated at 37°C for 2 h in the dark. The optical density was measured at 450 nm with a microplate reader. Cell viability (%) = OD value of experimental group/OD value of non-drug group×100%. The experiment was repeated three times independently.

### ELISA

The cell supernatant after drug intervention was discarded, and the cells were incubated in a 5.6 mmol/L glucose medium for 1 h. Then, the supernatant was taken, and the basal insulin secretion (BIS) levels were measured by ELISA using an insulin ELISA kit (Thermo Fisher, USA). The cells were incubated in a 30 mmol/L glucose medium for 1 h, and the supernatant was taken. GSIS levels were determined by ELISA. The total protein of each well was extracted and expressed as unit mass insulin (insulin content per well/corresponding protein content).

### qPCR

Total cell RNA was extracted using a TRIZOL RNA extraction kit (Invitrogen Inc., Carlsbad, CA, USA), followed by RT-PCR amplification after reverse transcription. The primers were designed with Primer 5 software and synthesized by Beijing Liuhe Huada Gene Technology Co., Ltd. (Beijing, China). The sequences are shown in Table 1. The PCR conditions were 1) pre-denaturation at 95°C for 1 min, and 2) denaturation at 95°C for 15 s, annealing at 63°C for 25 s, and extension at 72°C for 25 s, for 40 cycles. Statistical analysis was performed based on the ΔCt value, and the average relative expression (2^−ΔΔCt^) represented the relative amount between groups.

**TABLE 1:** Primers for real-time PCR.

Gene name	Forward primer (5′-3′)	Reverse primer (5′-3′)
GPR40	GGG​CAT​CAA​CAT​ACC​CGT​GAA	GCC​CTG​AGC​TTC​CGT​TTG​T-3′
IP3R-1	CGT​TTT​GAG​TTT​GAA​GGC​GTT​T	CAT​CTT​GCG​CCA​ATT​CCC​G-3′
GAPDH	AGG​GCT​GCC​TTC​TCT​TGT​GAC	TGG​GTA​GAA​TCA​TAC​TGG​AAC​ATG​TAG

### Western blot

Pancreatic tissue and RIN-m5f cells were lysed in NP-40 lysis buffer (FNN0021, Invitrogen Inc., Carlsbad, CA, USA) and centrifuged for 20 min (12,000 *×g*, 4°C). The protein concentration was measured using a BCA kit (Sigma, St. Louis, MO, USA). The proteins were separated by SDS-PAGE and transferred to PVDF membranes (Millipore Corp., Billerica, MA, USA). The membrane was blocked with 5% nonfat milk powder for 1 h at room temperature. Primary anti-rabbit monoclonal anti-GPR40 (32 kDa, 1:1000, SAB Biotherapeutics, Sioux Falls, SD, USA), anti-IP3R (314 kDa, 1:1000, Abcam, Cambridge, United Kingdom) and anti-GAPDH (36 kDa; 1:2000, Abcam, Cambridge, United Kingdom.) were incubated overnight at 4°C. The membranes were incubated with HRP-conjugated secondary antibody goat anti-rabbit (1:5000; Alexa Fluo, A21206:1723019; Thermo Fisher Scientific, Waltham, MA, USA) for 1 h at room temperature. The bands were visualized using enhanced chemiluminescence in the VersaDoc 4000 MP system (Bio-Rad, Hercules, CA, USA). Optical density values were analyzed using the Image-Pro Plus 6.0 software (National Institutes of Health, https://imagej.nih.gov/ij/) to determine the relative expression levels of GPR40 and IP3R proteins (Sharma et al., 2020).

### Statistical analysis

All data were shown as mean ± standard deviation. One-way ANOVA was used for comparison among multiple groups, with the least significant difference (LSD) *post hoc* test (consistent variance) or Dunnett’s-T3 *post hoc* test (uneven variance). *p* < 0.05 was considered statistically significant. SPSS 23.0 (IBM, Armonk, NY, USA) was used for statistical analysis. All graphs were drawn using Prism 6.02 (GraphPad Software Inc., San Diego, CA, USA) and Illustrator CS6 (Adobe Systems, San Jose, CA, USA).

## Results

### Principal component analysis of YD

Nine compounds were analyzed to obtain the characteristic spectra: salvianic acid A (characteristic component of Salviae Miltiorrhizae Radix Et Rhizoma), protocatechualdehyde (characteristic component of Salviae Miltiorrhizae Radix Et Rhizoma), chlorogenic acid (characteristic component of Atractylodis Rhizoma), puerarin (characteristic component of Puerariae Lobatae Radix), calycosin-7-O-β-D-glucopyranoside (characteristic component of Astragali Radix), salvianolic acid B (characteristic component of Salviae Miltiorrhizae Radix Et Rhizoma), atractylenolide I (characteristic component of Atractylodis Rhizoma), astragaloside IV (characteristic component of Astragali Radix) and astragaloside II (characteristic component of Astragali Radix) were used for quantitative and qualitative analysis.The contents of salvianolic acid A, protocatechuic aldehyde, chlorogenic acid, puerarin, verbascoside-7-O-β-D-glucopyranoside, salvianolic acid B, atractylodes I, astragaloside IV, astragaloside IV in YD were 0.32 mg/g, 0.35 mg/g, 0.10 mg/g, 0.34 mg/g, 0.14 mg/g, 0.35 mg/g, 0.11 mg/g, 0.10 mg/g and 0.11 mg/g (Table 2).

**TABLE 2:** Compounds in Yiqi Huazhuo decoction.

No.	Rt (min)	Molecular formula	Negative				Positive				Compounds
			Ion model	Measured mass (Da)	Calculated mass (Da)	Error (ppm)	Ion model	Measured mass (Da)	Calculated mass (Da)	Error (ppm)	
1	4.27	C_9_H_10_O_5_	[M-H]^-^	197.04456	197.04445	0.559	[M+H]^+^	199.06003	199.06010	−0.351	Salvianic acid A
2	6.43	C_7_H_6_O_3_	[M-H]^-^	137.02296	137.02332	−2.631	—	—	—	—	Protocatechualdehyde
3	9.72	C_16_H_18_O_9_	[M-H]^-^	353.08783	353.0867	3.176	[M+H]^+^	355.10205	355.10236	−0.869	Chlorogenic acid
4	13.06	C_21_H_20_O_9_	[M-H]^-^	415.10339	415.10236	2.485	[M+H]^+^	417.11743	417.11801	−1.387	Puerarin
5	17.16	C_22_H_22_O_10_	[M-H]^-^	445.11377	445.11292	1.902	[M+H]^+^	447.12814	447.12857	−0.969	Calycosin-7-O-β-D-glucopyranoside
6	22.31	C_36_H_30_O_16_	[M-H]^-^	717.1463	717.1463	1.797	[M+Na]^+^	741.14209	741.14260	−0.696	Salvianolic acid B
7	34.09	C_15_H_18_O_2_	—	—	—	—	[M+H]^+^	231.13779	231.13795	−0.720	Atractylenolide I
8	40.30	C_41_H_68_O_14_	[M+HCOO]^-^	829.45966	829.45801	1.987	[M+Na]^+^	807.44965	807.45013	−0.592	Astragaloside IV
9	40.55	C_43_H_70_O_15_	[M+HCOO]^-^	871.46997	871.46857	1.599	[M+Na]^+^	849.45990	849.46069	−0.933	Astragaloside II

### YD decreases the glucose and lipid levels and increases GPR40 in T2DM rats

**FIGURE 2:**
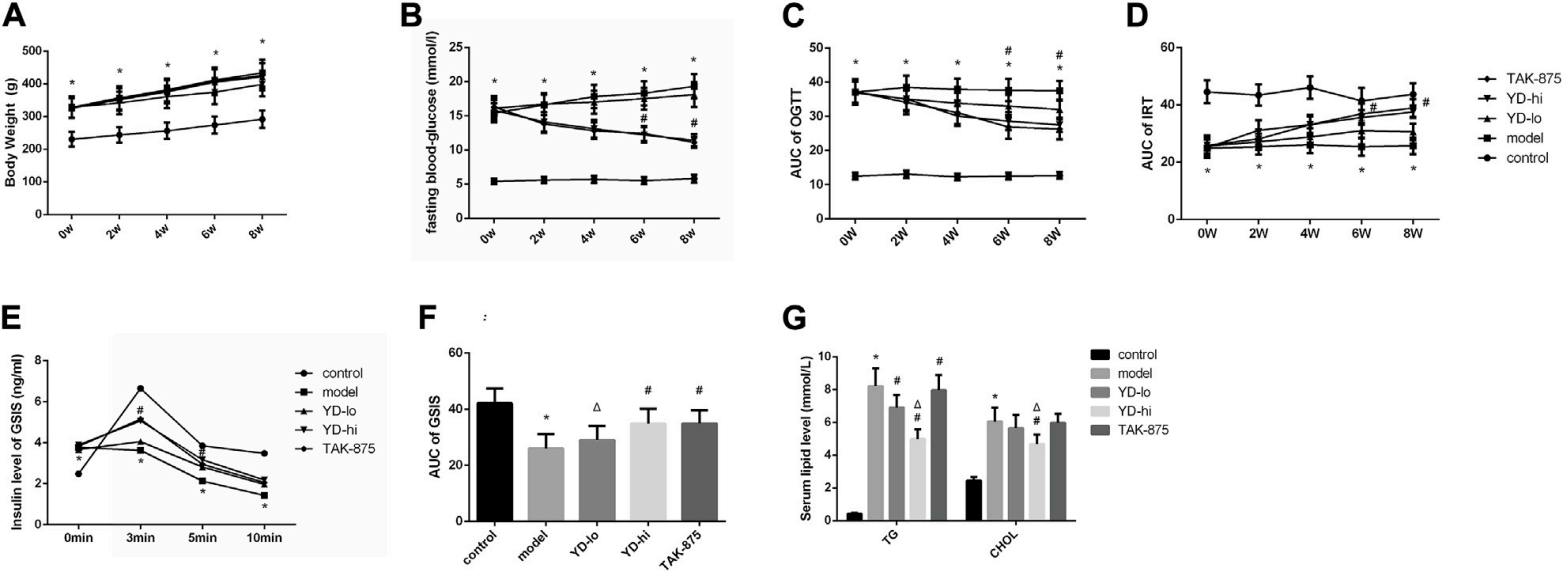
**(A)** Body weight of the rats after modeling. **(B)** Fasting blood glucose after modeling. **(C)** The area under the curve (AUC) of the glucose oral tolerance test (OGTT) after modeling. **(D)** The AUC of the insulin release test (IRT) after modeling. **(E)** Insulin levels of glucose-stimulated insulin secretion (GSIS) after modeling. **(F)** AUC of GSIS. **(G)** Triglycerides (TG) and total cholesterol (CHOL) levels. *n* = 8/group). ∗*p* < 0.05 vs. control group; ^#^ *p* < 0.05 vs. model group; ^Δ^ *p* < 0.05 vs. the TAK-875 group (positive control group).

After 8 weeks of treatment, the body weight of the rats in the model group increased from 141.3% of the control group to 148.8%, and the weight of the YD-hi group was 136.8% of the control group. The body weight of the YD-hi group was significantly lower compared with the model group (*p* < 0.05). The YD-lo and TAK-875 groups (positive control) were similar to the model group (*p* > 0.05). Hence, YD appeared to prevent weight gain in the YD-hi group (Figure 2A; Table 3). Moreover, the FBG of the rats in the model group increased from 287.3% of the control group to 332.8% after 8 weeks of treatment, while the YD-hi and TAK-875 groups were 196.6% and 191.4% of the control group, respectively. The FBG of the YD-hi and TAK-875 groups were reduced compared with the model group (*p* < 0.05). The YD-lo group was similar to the model group. These results indicated that YD-hi and TAK-875 could reduce FBG in rats (Figure 2B; Table 4).

**TABLE 3:** Body weight of the rats after modeling.

	0W	2W	4W	6W	8W
control	230.40 ± 22.14	243.41 ± 23.41	256.20 ± 25.22	274.10 ± 26.23	291.57 ± 26.45
model	327.42 ± 31.12∗	356.42 ± 34.52∗	381.55 ± 35.23∗	411.46 ± 38.61∗	433.51 ± 41.45∗
YD-lo	325.56 ± 30.45	351.39 ± 34.62	375.48 ± 36.11	405.41 ± 39.18	421.52 ± 41.27
YD-hi	328.62 ± 32.45	341.58 ± 34.29^#^	360.69 ± 35.32^#Δ^	374.41 ± 36.15^#Δ^	398.52 ± 37.21^#Δ^
TAK-875	327.51 ± 31.76	357.25 ± 35.37	376.41 ± 36.57	408.42 ± 40.62	425.57 ± 38.18

*n* = 8/group. ∗*p* < 0.05 vs. control group; ^#^ *p* < 0.05 vs. model group; ^Δ^ *p* < 0.05 vs. the TAK-875 group (positive control group).

**TABLE 4:** Fasting blood glucose after modeling.

	0W	2W	4W	6W	8W
control	5.41 ± 0.46	5.63 ± 0.53	5.54 ± 0.57	5.61 ± 0.62	5.87 ± 0.66
model	15.43 ± 1.44*	16.17 ± 1.96*	17.81 ± 1.78*	18.3 ± 1.89*	19.3 ± 2.02*
YD-lo	16.14 ± 1.65	16.61 ± 2.07	17.01 ± 1.67	17.56 ± 1.84	18.07 ± 2.09
YD-hi	15.97 ± 1.59	14.37 ± 2.18	13.27 ± 1.94^#^	12.25 ± 1.42^#^	11.75 ± 0.98^#^
TAK-875	16.24 ± 1.61	13.98 ± 1.95	12.78 ± 1.21^#^	12.41 ± 1.35^#^	11.17 ± 1.06^#^

*n* = 8/group. *p < 0.05 vs. control group; ^#^ *p* < 0.05 vs. model group; ^Δ^p < 0.05 vs. the TAK-875 group.

After 8 weeks of treatment, it was observed that the blood glucose levels (AUC value of OGTT) of the YD-lo, YD-hi, and TAK-875 groups were all decreased compared with the model group after 8 weeks of treatment (*p* < 0.05), while the blood glucose levels in the YD-hi and TAK-875 groups were decreased more than in YD-lo group, and these two groups were similar (*p* > 0.05) (Figure 2C; Table 5).

**TABLE 5:** The area under the curve (AUC) of the glucose oral tolerance test (OGTT) after modeling.

	0W	2W	4W	6W	8W
control	12.54 ± 1.06	12.35 ± 1.34	12.61 ± 2.07	12.62 ± 1.31	12.71 ± 1.24
model	37.15 ± 3.41∗	38.06 ± 3.52^∗^	37.94 ± 3.67∗	37.79 ± 4.05^∗^	37.58 ± 4.33^∗^
YD-lo	37.05 ± 3.62	35.14 ± 3.91	33.84 ± 3.54^#^	32.77 ± 4.19^#Δ^	32.17 ± 3.56^#Δ^
YD-hi	36.85 ± 3.54	35.12 ± 3.81	30.07 ± 2.85^#^	28.64 ± 2.61^#^	27.51 ± 2.49^#^
TAK-875	37.29 ± 1.67	34.10 ± 1.45	31.12 ± 2.57^#^	26.87 ± 1.95^#^	26.34 ± 1.82^#^

*n* = 8/group. ∗*p* < 0.05 vs. control group; ^#^ *p* < 0.05 vs. model group; ^Δ^p < 0.05 vs. the TAK-875 group.

The levels of insulin release (AUC value of IRT) in the YD-lo, YD-hi, and TAK-875 groups were increased compared with the model group after 8 weeks of treatment (*p* < 0.05), while the insulin release levels in the YD-hi and TAK-875 groups were higher than in YD-lo group, and these two groups were similar (*p* > 0.05) (Figure 2D; Table 6).

**TABLE 6:** The AUC of the insulin release test (IRT) after modeling.

	0W	2W	4W	6W	8W
control	44.65 ± 4.03	43.43 ± 3.75	46.13 ± 3.95	41.44 ± 4.62	43.73 ± 3.86
model	24.93 ± 2.75∗	25.47 ± 2.67∗	26.13 ± 2.95∗	25.45 ± 3.21∗	25.74 ± 2.86∗
YD-lo	25.65 ± 2.66	27.12 ± 2.81	28.84 ± 3.44	31.03 ± 2.77^#Δ^	30.61 ± 2.95^#Δ^
YD-hi	25.89 ± 3.21	28.14 ± 3.30	33.05 ± 3.23^#^	35.64 ± 2.61^#^	37.57 ± 2.14^#^
TAK-875	25.17 ± 2.64	31.12 ± 3.54	33.19 ± 3.41^#^	36.88 ± 3.58^#^	38.93 ± 3.13^#^

*n* = 8/group. ∗*p* < 0.05 vs. control group; ^#^ *p* < 0.05 vs. model group; ^Δ^ *p* < 0.05 vs. the TAK-875 group.

The GSIS AUC values in the YD-hi and TAK-875 groups were 133.9% and 134.2% of the model group, respectively, which were significantly higher than in the model group (*p* < 0.05); the YD-lo group was 106.4% of the model group (Figure 2F). The insulin secretion levels after glucose stimulation in the YD-hi, YD-lo, and TAK-875 groups were higher than in the model group at 3 and 5 min (all *p* < 0.05), while those in the YD-lo group were lower than in the YD-hi and TAK-875 groups. The insulin secretion levels in the YD-hi and TAK-875 groups were close (*p* > 0.05) (Figure 2E; Table 7). Hence, compared with the control group, the model group showed higher glucose levels and lower insulin secretion (all *p* < 0.05), while YD decreased glucose and increased insulin levels (all *p* < 0.05). YD-hi had similar effects to TAK-875 (*p* > 0.05).

**TABLE 7:** Insulin levels of glucose-stimulated insulin secretion (GSIS) after modeling.

	0min	3min	5min	10min
Control	2.48 ± 0.36	6.65 ± 0.64	3.85 ± 0.93	3.48 ± 0.88
Model	3.78 ± 1.02∗	3.63 ± 0.97∗	2.13 ± 0.89∗	1.43 ± 0.43∗
YD-lo	3.67 ± 0.75	4.06 ± 0.81^Δ^	2.82 ± 0.46^#^	3.17 ± 0.57^#Δ^
YD-hi	3.89 ± 0.67	5.07 ± 0.84^#^	3.17 ± 0.59^#^	2.18 ± 0.36^#^
TAK-875	3.83 ± 0.72	5.15 ± 0.78^#^	2.95 ± 0.42^#^	2.06 ± 0.34^#^

n = 8/group. ∗*p* < 0.05 vs. control group; ^#^ *p* < 0.05 vs. model group; ^Δ^ *p* < 0.05 vs. the TAK-875 group.

The serum TG and CHOL levels in the YD-hi group were decreased at 60.8% and 77.4% of the model group, respectively (*p* < 0.05). The TG levels in the YD-lo and TAK-875 groups were 88.6% and 96.7% of the model group, respectively, higher than in the YD-hi group (*p* < 0.05). The CHOL levels in the YD-lo and TAK-875 groups were 93.4% and 98.8% of the model group, respectively, which were also higher than in the YD-hi group (*p* < 0.05) (Figure 2G).

**FIGURE 3:**
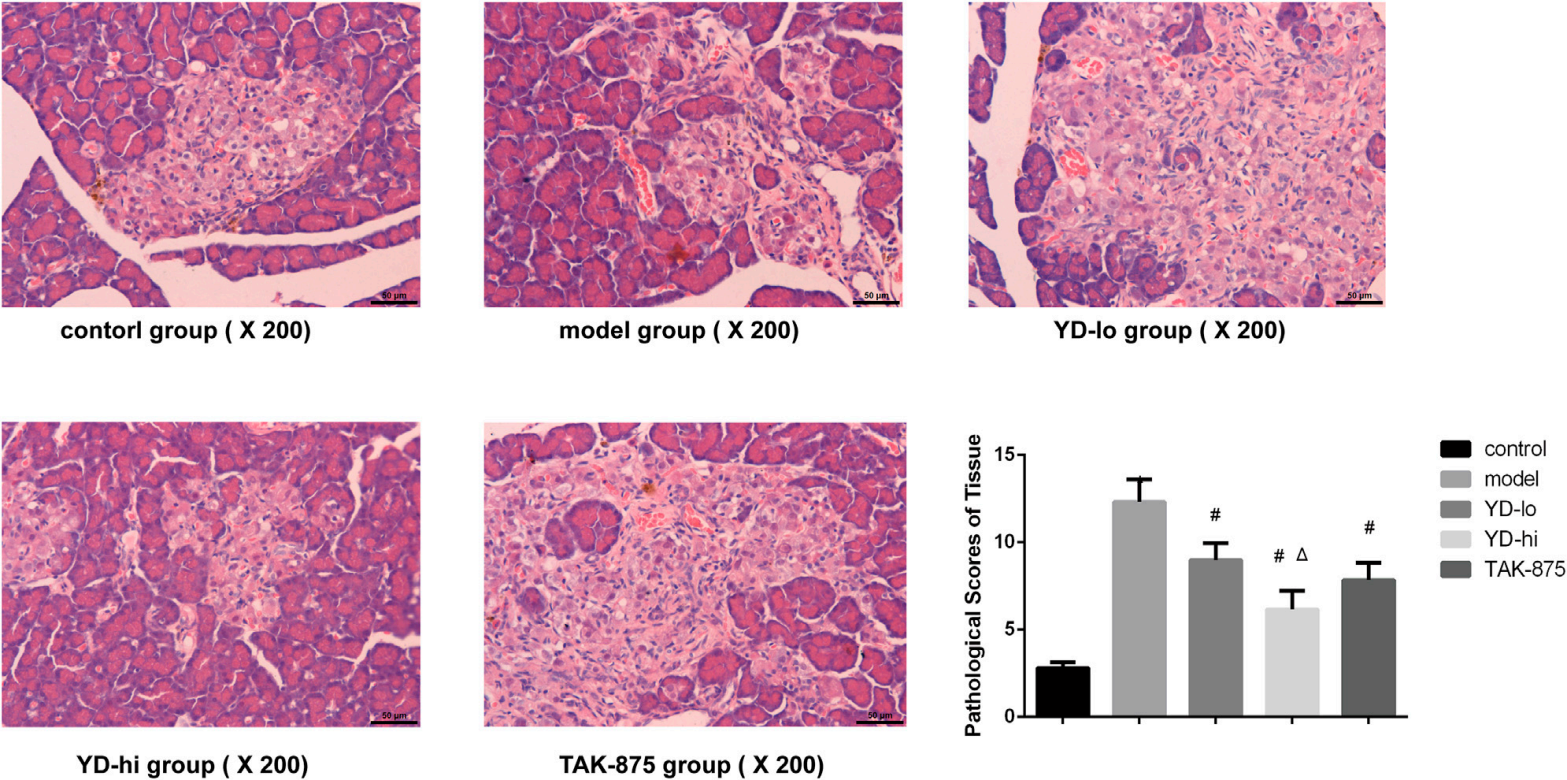
Histological examination and pathological scores of the pancreas in each group. *n* = 3/group. ∗*p* < 0.05 vs. the control group; ^#^ *p* < 0.05 vs. model group; ^Δ^ *p* < 0.05 vs. the TAK-875 group (positive control group). The yellow arrow indicates the cell nucleus.

Histopathological observation of the pancreas in the control group showed that the structure of the pancreas in the control group was normal, the cell morphology was intact, and no obvious pathological changes were found. In the model group, the acinar cells in the pancreatic tissue of the model group were normal, there were flaky fat deposits between the acinar lobules, and acinar cell hyperplasia and telangiectasia were observed in the pancreatic islets, and the pathology scores were higher than those in the control group (*p* < 0.05). A small amount of fat deposition was observed in the YD-hi group, and no acinar cell hyperplasia or telangiectasia was found. Partial fat deposition was seen in the YD-lo and TAK-875 groups, and a small amount of acinar cell dysplasia and telangiectasia were found. The pathological scores of YD-lo, YD-hi and TAK-875 groups were lower than those of the model group (*p* < 0.05), while those of YD-hi group were lower than those of TAK-875 group (*p* < 0.05) (Figure 3).

**FIGURE 4:**
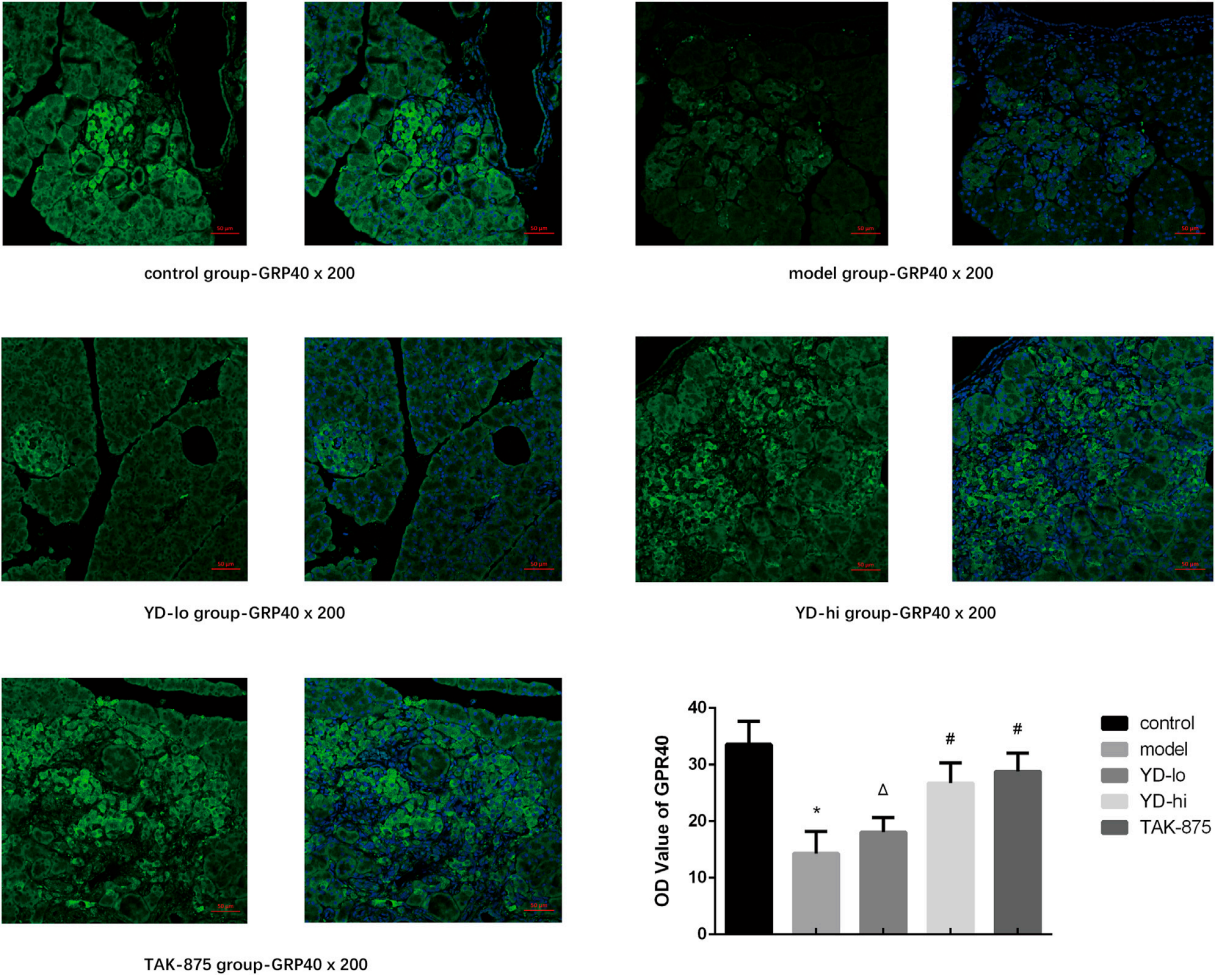
Fluorescence microscopy for the GPR40 protein. *n* = 3/group. ∗*p* < 0.05 vs. the control group; The GPR40 protein was shown in green fluorescence and the nucleus was shown in blue fluorescence. ^#^ *p* < 0.05 vs. model group; ^Δ^ *p* < 0.05 vs. the TAK-875 group (positive control group).

The GPR40 protein was observed using immunofluorescence (Figure 4). The fluorescence in the control group was the strongest, and the fluorescence in the YD-hi and TAK-875 groups was slightly weaker than in the control group, accounting for 79.6% and 85.8%, respectively (*p* < 0.05). The YD-lo and model groups showed the weakest GPR40 expression, accounting for 53.8% and 42.6% of the control group, respectively (*p* < 0.05). There were no significant differences between the YD-lo and YD-hi groups. Therefore, the increased insulin levels observed in the YD-hi group might be due to a higher expression of GPR40.

### Screening of YD concentration and intervention time in cells

**FIGURE 5:**
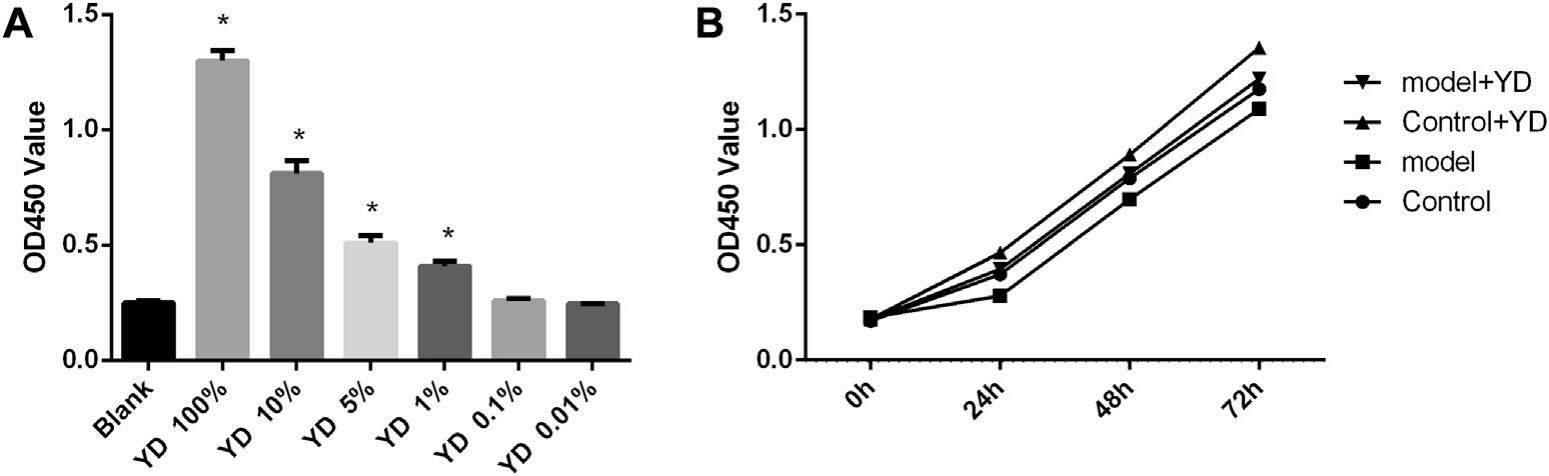
**(A)** Drug concentration screening. **(B)** Drug intervention time screening. *n* = 8/group). ∗*p* < 0.05 vs. control group.

The results of drug concentration screening showed that 100% YD had the strongest influence on cell proliferation, with YD 10%, 5%, and 1% also having an effect (all *p* < 0.05), but YD at 0.1% and 0.01% had no effect (*p* > 0.05) (Figure 5A). Because the YD components could interfere with optical density measurement, 10%, 5%, and 1% YD were selected for the subsequent experiments. The original decoction concentration was 3 g/mL; therefore, the 1% dose was 30 mg/mL, and the 5% dose was 150 mg/mL. Then, 1% YD was selected for drug intervention time screening, and the results showed that the cell survival rates at 48 and 72 h were better than at 24 h. Although the density of cells in the 72-h group was higher, the cell state was far inferior to that in the 48-h group (Figure 5B). Therefore, 48 h was selected.

### YD increases insulin secretion, GPR40, and IP3R-1 gene and protein expression in RINm5f cells

**FIGURE 6:**
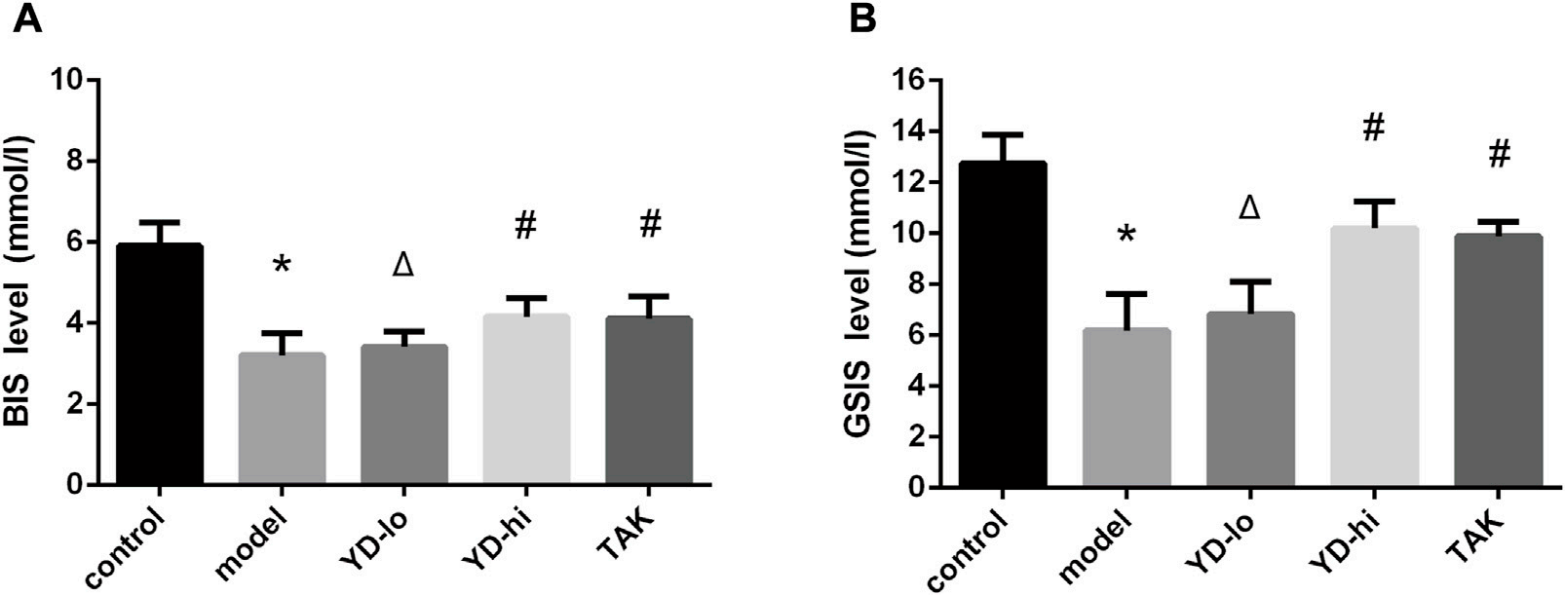
**(A)** Basal insulin secretion (BIS). **(B)** Glucose-stimulated insulin secretion (GSIS) test. ∗*p* < 0.05 vs. control group; ^#^ *p* < 0.05 vs. model group; ^Δ^ *p* < 0.05 vs. the TAK-875 group (positive control group).

The BIS and GSIS levels of the YD-hi group (150 mg/mL YD) were 124.5% and 178.3% of the model group, respectively (*p* < 0.05), and were 123.1% and 172.6% of the model group in the TAK-875 group, respectively (*p* < 0.05), without significant differences between the YD-hi and TAK-875 groups. The levels of BIS and GSIS in the YD-lo (30 mg/mL YD) group were not significantly different from the model group (*p* > 0.05) (Figures 6A, B).

**FIGURE 7:**
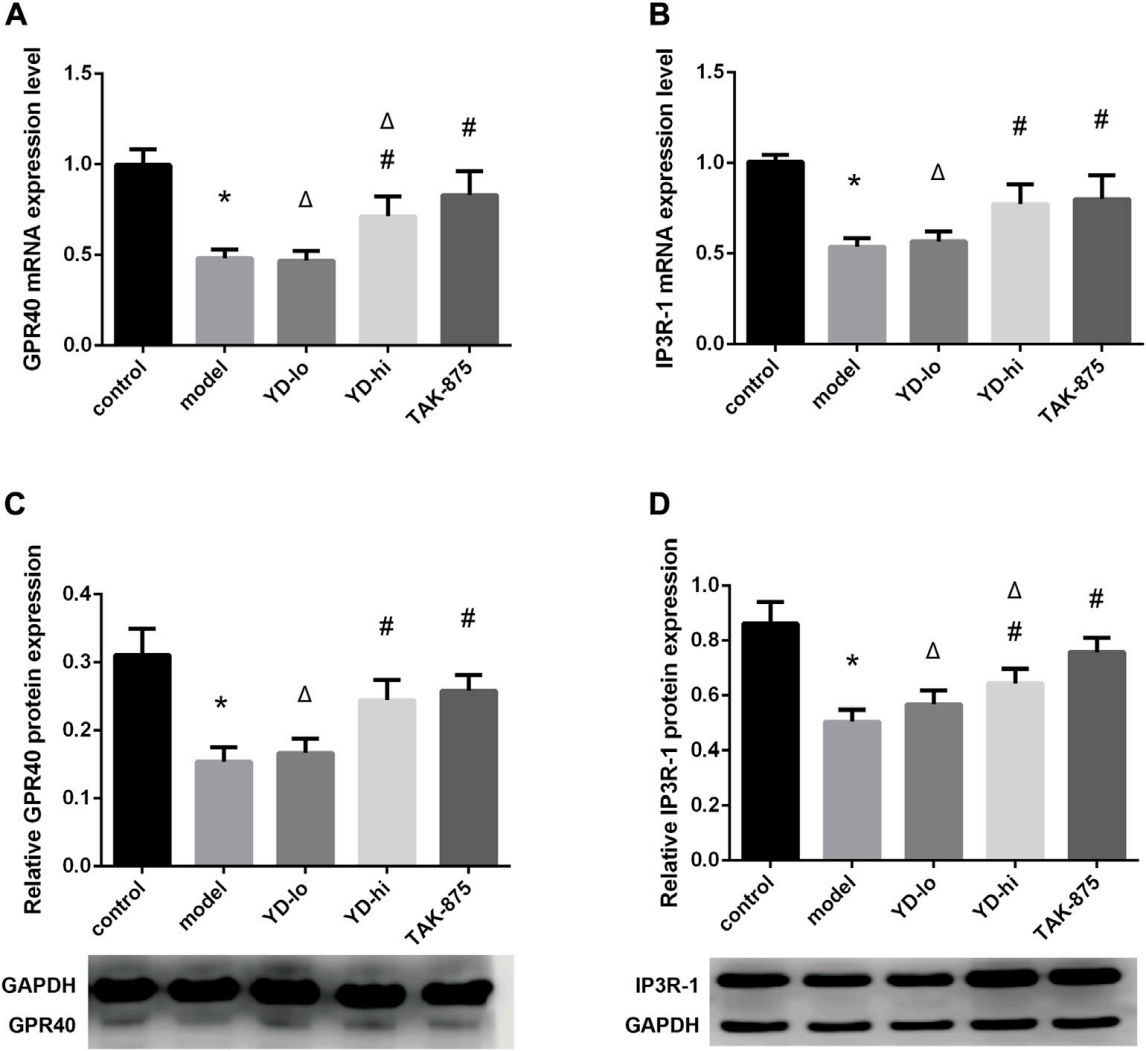
**(A)** GPR40 mRNA expression levels. **(B)** IP3R-1 mRNA expression levels. **(C)** GPR40 protein expression levels. **(D)** IP3R-1 protein expression levels. *n* = 8/group). ∗*p* < 0.05 vs. control group; ^#^ *p* < 0.05 vs. model group; ^Δ^ *p* < 0.05 vs. the TAK-875 group (positive control group).

The relative expression levels of the GPR40 mRNA in the YD-hi and TAK-875 groups were higher than in the model group (*p* < 0.05), the YD-lo group was close to the model group (*p* > 0.05), and the TAK-875 group was higher than the YD-hi group (*p* < 0.05) (Figure 7A). The protein expression levels of GPR40 were similar to that of mRNA, but there were no significant differences between the TAK-875 and YD-hi groups (*p* > 0.05) (Figure 7C). The relative expression of IP3R-1 mRNA in the YD-hi and TAK-875 groups was higher than in the model group (*p* < 0.05). There were no differences between the YD-lo and model groups. There were no differences in mRNA expression between the YD-hi and TAK-875 groups (Figure 7B). The trend of IP3R-1 protein expression was close to the mRNA expression but was higher in the TAK-875 group than in the YD-hi group (*p* < 0.05) (Figure 7D).

## Discussion

YD reduces blood glucose, glycated hemoglobin, body weight, and insulin resistance in patients with T2DM (Chen et al., 2013), but the exact mechanisms are unknown. Therefore, this study aimed to investigate the therapeutic effects and mechanism of YD on impaired insulin secretion in T2DM rats. The results indicated that YD promotes insulin secretion from pancreatic islet β-cells in T2DM rats by regulating the GPR40-IP3R-1 pathway, thereby reducing blood glucose. These results provide, at least in part, how YD increases insulin and decreases glucose levels in T2DM.

In a previous study, YD was found to significantly improve blood glucose, blood lipids, and body weight in patients with T2DM (Chen et al., 2013). YD also improved glucose tolerance and early-phase insulin secretion in T2DM rats and reduced body weight (Gong et al., 2013). Its safety has been proven in long-term clinical use and animal experiments (Chen et al., 2013; Gong et al., 2013). In the present study, different doses of YD were tested (50, 30, 15, and 5 g/kg) on T2DM rats, and 30 g/kg was the best dose for blood glucose control. No animals died during the study, and no obvious physical impairment or ill effects were observed. The CCK-8 assay in RIN-m5f cells also showed that YD had no obvious toxic effects on pancreatic β cells *in vitro*.

The effects of YD, a natural plant compound decoction, were investigated on blood glucose, blood lipids, insulin secretion, and pancreatic GPR40 expression in T2DM rats. YD reduced blood glucose, improved glucose tolerance, and improved insulin secretion in the first phase of T2DM rats and partially restored the decreased expression of pancreatic GPR40 induced by high glucose and high fat. The effect of a high concentration of YD was close to that of the GPR40 agonist TAK-875. These results were supported by Nagasumi et al. (Nagasumi et al., 2009), who found that transgenic mice with increased GPR40 gene expression had increased insulin secretion and glucose tolerance. Since GPR40 is a transmembrane protein specifically distributed in the pancreatic islet β-cell membrane, pancreatic tissue can be observed by confocal microscopy, and its protein expression can be calculated by measuring the optical density level. Unfortunately, the downstream IP3R-1 protein cannot be verified by immunohistochemistry, immunofluorescence, or western blot because it is not specific to β-cells and is widely expressed in the whole pancreas. Therefore, the RINm5f cell line had to be used to study IP3R-1.

*In vitro*, the BIS of RINm5f rat pancreatic β cells and the early-phase insulin secretion function after glucose stimulation were impaired to varying degrees after high-glucose and high-fat injury, and intracellular GPR40 and IP3R-1 expressions were both decreased, as previously observed (Robertson, 2009; Cerf, 2013). In the present study, YD could restore the insulin secretion function of cells to a certain extent, and the expression levels of GPR40 and IP3R-1 were increased to varying degrees. The effect of a high concentration of YD was similar to the positive control drug TAK-875, a drug already known for its effects on GPR40, glucose, and insulin in β-cells (Yashiro et al., 2012; Kaku et al., 2016). It indicated that the YD mixture could improve the damage of islet β cells caused by high-glucose and high-fat environments and promote basal insulin secretion and early-phase insulin secretion after glucose stimulation. The results of the *in vivo* and *in vitro* experiments were consistent.

Therefore, it could be hypothesized that high-glucose and high-fat injury inhibits the expression of GPR40 in the pancreatic islet β cell membrane, inhibiting the signal transduction of the GPR40-IP3R-1 pathway, resulting in decreased insulin secretion by β cells. YD could increase the expression of GPR40 and IP3R-1, leading to increased insulin secretion by islet β cells injured by high glucose and high fat.

This study had limitations. YD is a complex mixture of four herbs and several active compounds. Whether a single compound or the additive or synergistic effect of several compounds is responsible for the effects is unknown. Although YD was shown to increase GPR40 and IP3R-1, how YD increases GPR40 and IP3R-1 could not be determined in the present study. Future studies using knockdown and overexpression of the upstream and downstream proteins involved in GPR40 and IP3R-1 signaling will be necessary. YD could increase the insulin secretion of pancreatic β-cells and reduce blood glucose through the GPR40-IP3R-1 signaling pathway. It can be hypothesized that the GPR40-IP3R-1 signaling pathway plays an important role in the impaired insulin secretion and pathogenesis of T2DM.

## Data Availability

The original contributions presented in the study are included in the article/supplementary materials, further inquiries can be directed to the corresponding author/s.
